# Sediment Bacteria in the Alpine Lake Sayram: Vertical Patterns in Community Composition

**DOI:** 10.3390/microorganisms11112669

**Published:** 2023-10-30

**Authors:** Keqiang Shao, Boqiang Qin, Jianying Chao, Guang Gao

**Affiliations:** 1 State Key Laboratory of Lake Science and Environment, Nanjing Institute of Geography and Limnology, Chinese Academy of Sciences, Nanjing 210008, China; kqshao@niglas.ac.cn (K.S.); qinbq@niglas.ac.cn (B.Q.); 2 Ministry of Environmental Protection, Nanjing Institute of Environmental Sciences, Nanjing 210042, China

**Keywords:** Lake Sayram, carbonate, TOC, sediment, BCC, vertical distribution

## Abstract

Bacterial communities inhabiting alpine lakes are essential to our understanding of ecosystem processes in a changing climate, but little has been reported about the vertical patterns of sediment bacterial communities in alpine lakes. To address this knowledge gap, we collected the 100 cm long sediment core from the center of Lake Sayram, the largest alpine lake in Xinjiang Uygur autonomous area, China, and used 16S rRNA gene-targeted amplicon sequencing to examine the bacterial populations. The results showed that bacterial diversity, as estimated by the Shannon index, was highest at the surface (6.9849 at 0–4 cm) and gradually decreased with depth up to 3.9983 at 68–72 cm, and then increased to 5.0927 at 96–100 cm. A total of 56 different phyla and 1204 distinct genera were observed in the sediment core of Lake Sayram. The bacterial community structure in the sediment samples from the various layers was dissimilar. The most abundant phyla in alpine Lake Sayram were *Proteobacteria*, *Firmicutes*, and *Planctomycetes*, accounting for 73%, 6%, and 4% of the total reads, respectively; the most abundant genera were *Acinetobacter*, *Hydrogenophaga*, and *Pseudomonas*, accounting for 18%, 12%, and 8% of the total reads, respectively. Furthermore, the relative abundance of *Acinetobacter* increased with sediment depth, while the relative abundance of *Hydrogenophaga* and *Pseudomonas* decreased with sediment depth. Our findings indicated that the nitrate-reducing bacteria (*Acinetobacter*, *Hydrogenophaga*, and *Pseudomonas*) may be prevalent in the sediment core of Lake Sayram. Canonical correspondence analysis showed that carbonate and total organic carbon (TOC) may be the main environmental factors affecting the vertical patterns of bacterial community composition (BCC) in the sediment of Lake Sayram. This work significantly contributes to our understanding of the BCC of sediments from alpine lakes in arid and semiarid regions.

## 1. Introduction

Alpine lakes are an important source of water supply in arid and semiarid regions [1], and they are regarded as primitive ecosystems because they are remote, difficult to access, and undisturbed by human activities [2]. Alpine lakes are distinguished by extreme environmental circumstances such as cold temperatures, high UV radiation, and oligotrophic nature with low primary productivity [2]. These extreme conditions influence the lake hydrology and structure, as well as the BCC and biogeochemical functioning [3,4,5]. Since alpine lakes have been recognized as an early indicator of environmental change, the study of bacterial communities has received considerable attention [6].

Bacterial communities in sediments play an important role in the mineralization of nutrients and the breakdown of organic matter in alpine lakes [2,7]. They are believed to be a sensitive sentinel to environmental changes [8] and can serve as important indicators of water pollution and potential biomarkers for evaluating environmental stressors [9]. Therefore, understanding the sediment bacterial community is critical for accurately evaluating the ecosystem processes of alpine lakes [7]. Numerous earlier studies have investigated the bacterial diversity and community composition in alpine lakes, but they only focused on the winter cover [10], water column [11], and surface sediment [2,12,13]. Our knowledge of the vertical patterns of sediment BCC in alpine lakes, particularly those in arid and semiarid regions, is very limited.

Arid and semiarid regions cover nearly one third of the world’s land area, with China accounting for 52.5%. Lakes in Central Asia are an essential component of the regional water resource system, providing limited but vital water supplies for both the fragile environment and humans [14]. The Tianshan Mountains are located in Central Asia and stretch through Xinjiang, China, Kazakhstan, and Kirghizstan, with a total length of 2500 km running east–west, with widths ranging from 250 km to 400 km [15]. Lake Sayram, in China’s arid northwestern region, is an alpine lake in the heart of the Tianshan Mountains and the country’s largest deep lake. Previous study has investigated the prokaryotic diversity in the water of Lake Sayram [15]. However, little information is now available about the entire bacterial community inhabiting the sediments of this alpine lake.

Thus, this study was carried out to assess the bacterial community structure at various depths in the sediment for Lake Sayram. To accomplish this, sediment samples were assessed using Illumina MiSeq sequencing. Specifically, this study of Lake Sayram was conducted in order to (1) analyze the bacterial community structure of the sediment at various depths in Lake Sayram and (2) identify which main environmental factors govern the vertical patterns of sediment BCC.

## 2. Materials and Methods

### 2.1. Study Area and Sample Collection

Lake Sayram, China’s largest oligotrophic alpine lake in the Xinjiang Uygur autonomous area, is renowned as a “pearl” along the Silk Road [16]. The lake is 30 km from north to south and 27 km from east to west. The mean water temperature of the lake is 1.1 °C, and the highest temperature is roughly 19 °C; hence, the evaporation rate is relatively low. Water transparency ranges between 8 and 13 m, with a mean water depth of 46 m and a maximum depth of 90 m. On 16 August 2018, a 100 cm long sediment core was obtained from the lake’s center (44.597312° N and 81.206671° E) using a piston corer. The sediment core was subsampled in situ at 4 cm intervals, and these 25 wet sediment samples were transferred into sterile plastic containers and kept at −80 °C for storage until they were freeze-dried.

### 2.2. Chemical Analysis

The content of bulk carbonate (carb%) of sediments was determined via titration with diluted perchloric acid (HClO_4_; 0.1 mol L^−1^), with an analytical precision of better than 0.5%. The content of total nitrogen (TN; %) of sediments was analyzed using the micro-Kjeldahl method. The content of total organic carbon (TC; %) and carbon to nitrogen (C/N) ratio of sediments were measured with an elemental analyzer (Elementar, Frankfurt, Germany).

### 2.3. DNA Extraction and Illumina Sequence Processing and Analysis

Crude DNA from each sediment sample was extracted using the PowerClean^TM^DNAClean-Up Kit for Soil (Mo Bio, Carlsbad, CA, USA) according to the manufacturer’s procedures. The V3–V4 hypervariable regions of the bacterial 16S rRNA genes were amplified using the primer set 338F (5′-ACTCCTACGGGAGGCAGCAG-3′) and 806R (5′-GGACTACHVGGGTWTCTAAT-3′) [17]. Unique barcodes were placed into the primers for each sample. Polymerase chain reaction (PCR) amplification was carried out in a 50 mL reaction mixture, including 5 mL of 10 × PCR buffer, 4 mL of MgCl_2_ (25 mmol L^−1^), 0.5 mL of each primer (10 mmol L^−1^), 15 ng of quantified DNA template, and 0.4 mL of Taq polymerase (5 U mL^−1^; Fermentas, Waltham, MA, USA). A touchdown program was used to perform the PCR program in a thermocycler (Applied Biosystems Veriti Thermal Cycler, Waltham, MA, USA): denaturation at 94 °C for 5 min, 11 cycles of denaturation at 94 °C for 1 min, annealing at 60 °C for 1 min (temperature was decreased by 1 °C every cycle until 50 °C was reached), and extension at 72 °C for 1 min. Nineteen additional PCR cycles were carried out at an annealing temperature of 50 °C, followed by a final extension at 72 °C for 10 min. Then, three parallel PCR reactions were performed and pooled for each sample. The PCR products were purified with AMPure XP beads to eliminate PCR-induced biases. Agarose gel electrophoresis and a NanoDrop 2000 spectral photometer (Thermo Scientific, Waltham, MA, USA) were used to assess the quality and quantity of DNA. Equimolar amounts of barcoded amplicons for each sample were sequenced using the Illumina MiSeq PE300 platform of the Guangzhou Gene Denovo Biotechnology (Co., Ltd.) (Guangzhou, China).

The bioinformatic analysis was carried out on CLC Genomics Workbench 20.0 (Qiagen, Aarhus C, Denmark) with the Microbial Genomics Module. Following the import of raw reads, a standard quality control procedure was carried out that involved merging paired reads (minimum overlap of 200 bp) and trimming off adapters and primer sequences. A 97% similarity rate was used to assign microbial phylotypes to operational taxonomic units (OTUs). We removed low abundance OTUs (10 reads) to reduce the random sequencing error. Taxonomic classification was performed by comparing the reads against the SILVA small subunit rRNA (SSU) database v.132 [18]. Sequences associated with chimeras and chloroplasts were eliminated from further analysis.

The Mothur program (v.1.31.2) was used to assess the bacterial α-diversity indicators, including the Shannon, Simpson, Chao1, and ACE indices, after normalizing the sequencing depth, based on the sample with the smallest sequencing effort. Cluster analysis based on the Bray–Curtis distance was conducted to assess the bacterial β-diversity across sediment layers.

### 2.4. Statistical Analyses

To test the difference in the BCC among the 14 sediment samples, we performed cluster analysis using PERMANOVA with 999 permutations [19]. Canonical correspondence analysis (CCA) was used to analyze the relationships between the community structures of the different samples and sediment physiochemical properties. All data were log(x + 1)-transformed. The CCA was carried out using the CANOCO 5.0 software (Ithaca, NY, USA) using the unimodal method because detrended correspondence analysis run on species variables indicated that the length of the first axis was >4. The significance of the first ordination and canonical axes together was assessed in permutation tests with 499 unrestricted Monte Carlo permutations.

### 2.5. Nucleotide Sequence Accession Number

All raw sequencing reads have been deposited at the Sequence Read Archive database of the National Center for Biotechnology Information (NCBI, Bethesda, MD, USA) under accession number PRJNA853917.

## 3. Results

### 3.1. Sediment Chemical Properties

The depth distributions of nutrient content in the sediment core of Lake Sayram are shown in Table 1. The carbonate content (carb%) in the sediment core varied between 35.3% at the surface (0–4 cm) and 47.5% at 16–20 cm, with an average of 39.6%. The TOC content was lowest at the surface (4.15% at 0–4 cm) and was highest at 73–76 cm, with an average of 8.32%. The TN content was lowest at the surface (0.47% at 49–52 cm) and was highest at 37–40 cm, with an average of 0.60%. The C/N ratio was lowest at the surface (7.73 at 0–4 cm) and was highest at 73–76 cm, with an average of 15.05.

### 3.2. Vertical Changes in Bacterial α-Diversity

We generated 11,961,222 high-quality reads, with an average of 498,384 reads per sample. These readings were classified into 7029 OTUs across the 25 different sediment layer samples. After 37,500 reads, the rarefaction curves for Chao1 bias-corrected richness began to converge toward an asymptote, suggesting sufficient depth for further analysis. This was also supported by the high Good’s coverage, ranging from 98.39% to 99.85% (Table 2).

The change patterns of bacterial α-diversity indicators, including the Shannon, Simpson, Chao1, and ACE indexes, were distinct across the 25 different sediment layers of Lake Sayram. The Shannon and Simpson indexes were high at the surface (6.9849 at 0–4 cm and 0.9759 at 0–4 cm, respectively) and gradually decreased with depth up to 3.9983 and 0.7908 at 68–72 cm, respectively, and then increased to 5.0927 at 96–100 cm and 0.9157 at 92–96 cm, respectively. The Chao1 and ACE indexes were also higher at the surface (1366.60 at 0–4 cm and 1029.10 at 0–4 cm, respectively), steadily decreased at 80–84 cm, and finally increased to 898.17 and 610.84 at 96–100 cm, respectively (Table 2).

### 3.3. Vertical Distribution of the Bacterial Community

**Figure 1:**
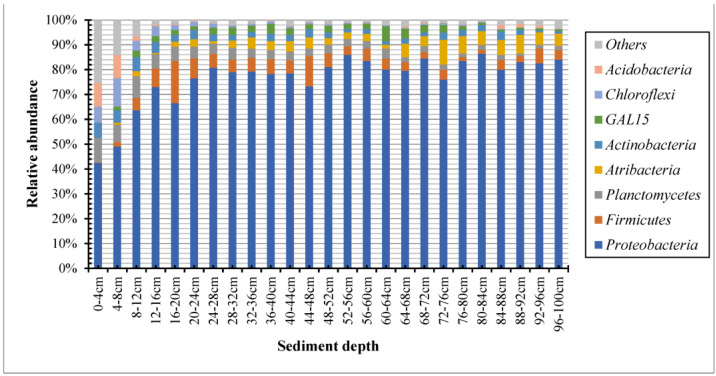
Relative abundance of bacterial community at the phylum level from different layers of sediments in Lake Sayram. Only phyla with mean relative abundance exceeding 1% are represented.

**Figure 2:**
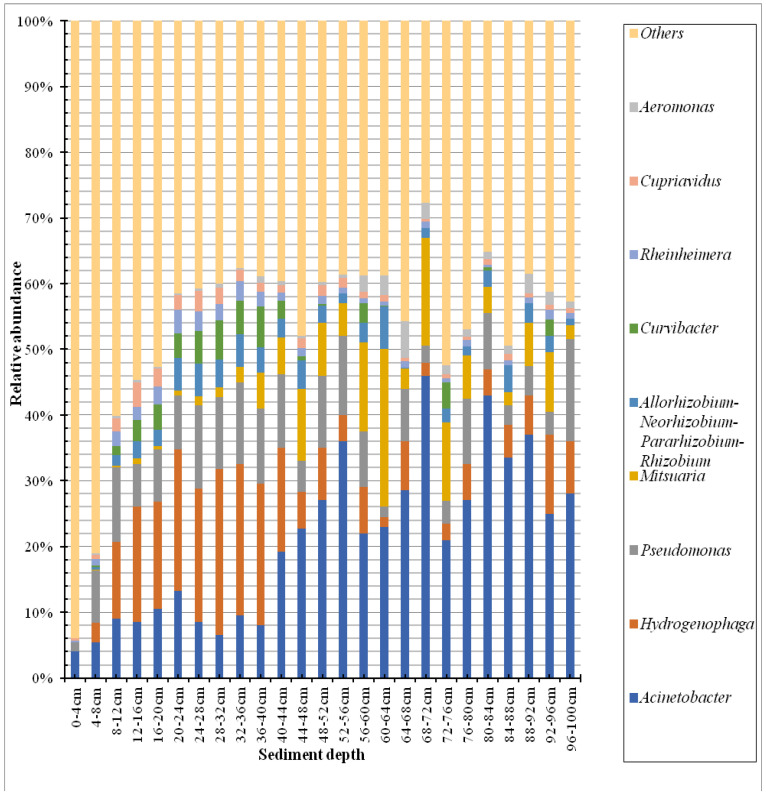
Relative abundance of bacterial community at the genus level from different layers of sediments in Lake Sayram. Only genera with mean relative abundance exceeding 1% are represented.

We detected a total of 56 different phyla of sediment core in Lake Sayram. The dominant phyla (mean relative abundance > 1%) were *Proteobacteria*, *Firmicutes*, *Planctomycetes*, *Atribacteria*, *Actinobacteria*, *GAL15*, *Chloroflexi*, and *Acidobacteria*, accounting for 76.41%, 5.08%, 3.77%, 3.76%, 2.79%, 2.21%, 1.60%, and 1.13% of the total reads, respectively (Figure 1). In addition, a total of 1204 different genera were obtained, and the dominant genera (mean relative abundance > 1%) were *Acinetobacter*, *Hydrogenophaga*, *Pseudomonas*, *Mitsuaria*, *Allorhizobium–Neorhizobium–Pararhizobium–Rhizobium*, *Curvibacter*, *Rheinheimera, Cupriavidus*, and *Aeromonas*, accounting for 20.89%, 10.17%, 7.97%, 5.67%, 2.77%, 1.94%, 1.45%, 1.37%, and 1.20% of the total reads, respectively (Figure 2).

**Figure 3:**
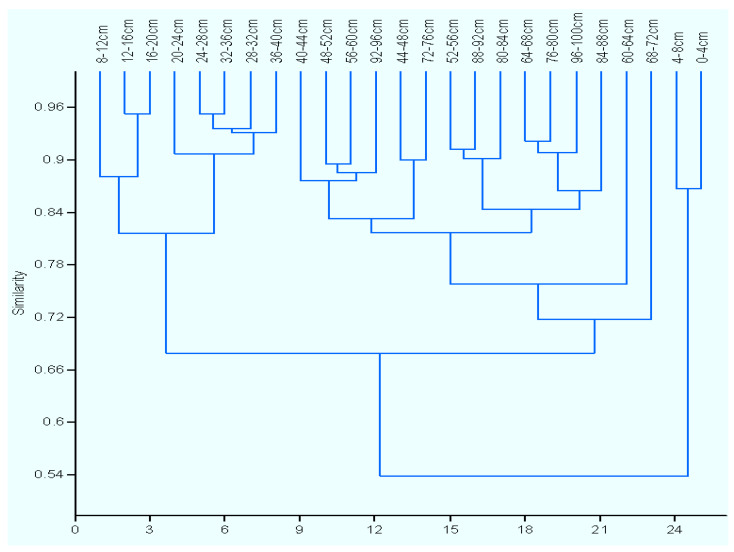
Cluster analysis dendrogram of the sediment BCC from different layers’ sediments in Lake Sayram.

Cluster analysis showed that the BCC values from the 25 layers of the sediment core of Lake Sayram were grouped into three different defined clusters (Figure 3). The first cluster contained layers from 0 to 8 cm, the layers in the second cluster ranged from 8 to 40 cm, and the third cluster included layers ranging from 40 to 100 cm. The UPGMA dendrogram revealed that the bacterial communities of the three clusters were dissimilar to each other in different degrees. The community structure of the sediments derived from 0–8 cm layers shared merely 54% similarity to the other two clusters, while 68% similarity was observed between the second and third clusters.

### 3.4. Main Environmental Factors Affecting Sediment BCC

**Figure 4:**
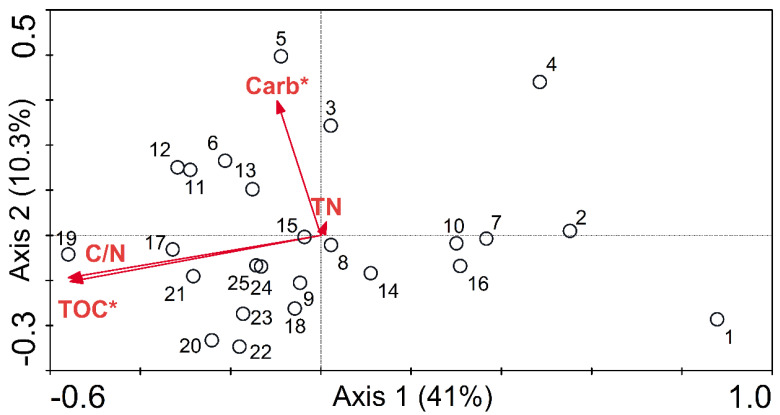
The CCA ordination plot suggests the relationships between the sediment BCC and environmental variables. According to a Monte Carlo permutation test (*p* < 0.05), the variable that is statistically significant is denoted with an asterisk (*).

We performed a CCA biplot analysis of the sediment bacterial communities and the four environmental factors (Carb, TOC, TN, and C/N) for Lake Sayram (Figure 4). The CCA biplot demonstrated that carbonate and TOC may be the main environmental factors influencing the vertical patterns of the sediment BCC for Lake Sayram. The eigenvalues of the first and second axes were 0.108 and 0.027, respectively, and these two axes explained 51.3% of the vertical variation in the sediment BCC for Lake Sayram.

## 4. Discussion

Microbial ecologists have long been interested in understanding the change patterns of bacterial communities across sediment depths. The knowledge of the vertical distribution of the BCC in sediments from alpine lakes has, however, received relatively little attention. In this study, we explored the vertical distribution of sediment bacterial communities in the alpine Lake Sayram, China. Our results showed that the bacterial community structure appeared to be relatively heterogeneous among the different layers of the sediment core, as indicated by the UPGMA dendrogram (Figure 3). However, there was obvious variation in the BCC among the sediment layers ranging from 0 to 8 cm, 8 to 40 cm, and 40 to 100 cm. Specifically, significant differences were found in the relative abundance of the most dominant subdivisions. Firstly, the relative abundance of *Proteobacteria* and *Firmicutes* in the sediment layers from 0 to 8 cm was lower than that of the sediment layers from 8 to 100 cm, but the relative abundance of *Planctomycetes* in the sediment layers from 0 to 8 cm was higher than that of the sediment layers from 8 to 100 cm. Second, the relative abundance of *Acinetobacter* in the sediment layers from 0 to 40 cm was lower than that of the sediment layers from 40 to 100 cm, but the relative abundance of *Hydrogenophaga* and *Pseudomonas* in the sediment layers from 0 to 8 cm was lowest, and the abundance was higher in layers from 8 to 40 cm than in sediment layers from 40 to 100 cm.

In the present study, *Proteobacteria* was the most abundant phylum, commonly observed in all layers of the sediment core from Lake Sayram. *Proteobacteria* was also found to be the most dominant bacterial group in water samples for Lake Sayram [15]. As the largest phylum of bacteria, *Proteobacteria* play an important role in the functioning and processes of lake ecosystems [20,21]. *Proteobacteria* has been suggested as a representative bacterial lineage in waters and sediments from other saline and alkaline lakes [22,23,24,25] and marine coastal waters [26]. They also have been observed to be the dominant bacterial group in the sediment of freshwater lakes [27,28]. The second dominant phylum in the sediment core of Lake Sayram was *Firmicutes*. They can be viewed as unique markers that are present only in soil with a high salinity rate and are not present in other hypersaline environments [23,29]. According to previous studies, *Firmicutes* was also found as the main phylum in subtropical paddy soils [30], in alkaline lake sediments across the Tibetan Plateau [10], and in saline soils in China [31]. In addition, studies in Antarctica’s arid and saline soils also indicated that *Firmicutes* was the phylum most significantly associated with salinity [32,33]. The third abundant phylum in the sediment core of Lake Sayram was *Planctomycetes*, and they have the ability to degrade complex sulfated polysaccharides of algal origin [34,35]. *Planctomycetes* are primarily distinguished by their attached lifestyle that involves adhering to surfaces in a variety of environments, including aquatic, harsh, and contaminated habitats [35]. They were also found as the major phylum in the saline–alkali soils of Songnen Plain, China [36].

A total of 1204 different genera were identified in our study, suggesting a higher bacterial diversity in the sediment core of Lake Sayram. Among these genera, *Acinetobacter*, *Pseudomonas*, and *Hydrogenophaga*, affiliated with *Proteobacteria*, are the most dominant genera. The extensive distribution and high abundance of *Acinetobacter* in the sediment are of interest because they have been frequently described as hydrogen-oxidizing autotrophic denitrifying bacteria in earlier studies [37,38]. *Acinetobacter* had a successional development with a decrease in temperature, as reported by Yong et al. [39]. This may explain why *Acinetobacter* is the most dominant genus in the sediment core of Lake Sayram, which is a cold-water inland lake, with a mean temperature of about 1.1 °C and a maximum temperature of roughly 19 °C [16]. Previous studies have also shown that many *Acinetobacter* species isolated from oligotrophic habitats have a stronger tolerance to low temperatures and can thrive well in cold environments [40,41,42].

Denitrification and dissimilatory nitrate reduction to ammonium (DNRA) are two competing processes for nitrate reduction [43]. Some species of *Pseudomonas* are facultative anaerobic heterotrophic denitrifying bacteria with excellent resistance to aromatic pollutant toxicity and high efficiency in completely decomposing a wide variety of aromatic chemicals [39]. Additionally, they also play an important role in wastewater treatment processes due to their high biodegradation ability for a variety of organic chemicals [39]. Some *Hydrogenophaga* species have been found to be important anaerobic DNRA bacteria [43] and potential degraders for petroleum hydrocarbon and polynuclear aromatic hydrocarbons (PAHs) [44]. A previous study has also linked *Pseudomonas* and *Hydrogenophaga* to hydrogen autotrophic denitrification [41]. In our investigation, it was clearly observed that the relative abundance of *Acinetobacter* increased with sediment depth (Figure 2), suggesting that a greater degree of hydrogen autotrophic denitrification occurred in the deeper sediment layers than in the top sediment layers. In addition, the relative abundance of *Hydrogenophaga* and *Pseudomonas* decreased with sediment depth, suggesting that the nitrate reduction activity may be stronger in the top layer of the sediment and may weaken with sediment depth. Unfortunately, we have no data on the source of nitrate and hydrogen from the sediments in this study.

According to the CCA findings of our investigation, carbonate and TOC may be the most significant environmental factors influencing the vertical patterns of bacterial communities in the sediment for Lake Sayram (Figure 4). A greater number of nutrients in aquatic sediment environments are likely to promote richer and more diverse bacterial communities through increased niche partitioning [45,46]. As a major nutrient for aquatic ecosystems, different types of carbon have been identified as the most critical nutrient-limiting factors for bacterial growth [47]. The importance of TOC in shaping the vertical changes in sediment bacterial communities has been found in eutrophic Lake Taihu, China [28]. Furthermore, according to a study by Bai et al. [47], TOC was also the primary environmental factor responsible for changes in the bacterial population in the sediment in another eutrophic lake (Dianchi) in China. However, close associations between bacterial communities and carbonate derivatives, such as calcium carbonate (CaCO_3_), were only observed in an area with acidic paddy soil in southern China [48], and in Karst caves in Italy [49], and have been rarely found in aquatic ecosystems. According to the findings of Guo et al. [48], applying low and moderate amounts of CaCO_3_ can increase soil bacterial diversity and modify the BCC. Conversely, microbes can enhance the dissolution of CaCO_3_ [49]. Furthermore, both autotrophic and heterotrophic bacteria, such as nitrogen-fixing bacteria, which hydrolyze urea and cellulose, play an important role in CaCO_3_ precipitation [48]. This may suggest that the nitrate-reducing bacteria (*Acinetobacter*, *Hydrogenophaga*, and *Pseudomonas*) are prevalent in the sediment core of Lake Sayram in this study, which can also explain their different changes with depth in the sediment.

## 5. Conclusions

In summary, Illumina MiSeq sequencing revealed substantial bacterial diversity and heterogeneous vertical zonation of bacterial community structure in the sediment for the alpine Lake Sayram, China. *Proteobacteria*, *Firmicutes*, and *Planctomycetes* were the most abundant phyla, accounting for 73%, 6%, and 4% of all reads, respectively, and the most abundant genera were *Acinetobacter*, *Hydrogenophaga*, and *Pseudomonas*, accounting for 18%, 12%, and 8% of the total reads, respectively. Furthermore, the relative abundance of *Acinetobacter* increased with sediment depth, while the relative abundance of *Hydrogenophaga* and *Pseudomonas* decreased with sediment depth. Our results also indicated that the nitrate-reducing bacteria (*Acinetobacter*, *Hydrogenophaga*, and *Pseudomonas*) may be prevalent in the sediment core of Lake Sayram. The CCA showed that carbonate and TOC concentrations were significantly correlated with the vertical changes in the sediment bacterial communities of Lake Sayram.

## Figures and Tables

**Table 1:** The carb, TOC, TN content, and C/N ratio in different layers of sediment core from Lake Sayram.

Sediment Depth	Carb%	TOC (%)	TN%	C/N
0–4 cm	35.000	4.150	0.537	7.734
4–8 cm	39.000	4.790	0.543	8.816
8–12 cm	44.000	5.780	0.533	10.836
12–16 cm	45.000	4.250	0.542	7.841
16–20 cm	47.000	6.140	0.568	10.816
20–24 cm	43.000	6.780	0.543	12.495
24–28 cm	39.000	4.850	0.497	9.760
28–32 cm	39.000	6.160	0.501	12.302
32–36 cm	38.000	6.930	0.553	12.523
36–40 cm	39.000	6.280	0.604	10.399
40–44 cm	43.000	7.250	0.572	12.672
44–48 cm	43.000	7.190	0.553	13.003
48–52 cm	41.000	6.300	0.466	13.517
52–56 cm	38.000	6.240	0.532	11.720
56–60 cm	39.000	6.140	0.466	13.174
60–64 cm	38.000	5.580	0.532	10.480
64–68 cm	39.000	7.400	0.494	14.968
68–72 cm	37.000	7.100	0.554	12.814
72–76 cm	40.000	8.320	0.553	15.049
76–80 cm	36.000	7.760	0.547	14.186
80–84 cm	38.000	7.420	0.503	14.757
84–88 cm	35.000	7.460	0.501	14.898
88–92 cm	37.000	7.450	0.553	13.463
92–96 cm	39.000	7.320	0.582	12.569
96–100 cm	39.000	7.280	0.573	12.702

**Table 2:** The OTU-based species diversity as represented by the Shannon, Simpson, Chao1, and ACE indices, as well as Good’s coverage, in different layers of sediment core from Lake Sayram.

Sediment Depth	Shannon	Simpson	Chao1	ACE	Good’s Coverage
0–4 cm	6.9849	0.9759	1366.5989	1029	0.9923
4–8 cm	6.5669	0.9681	1384.6646	1014	0.9912
8–12 cm	6.6422	0.9716	1383.3880	1024	0.9943
12–16 cm	6.2423	0.9696	1062.9475	810	0.9956
16–20 cm	5.7857	0.9507	900.5745	693	0.9839
20–24 cm	5.8550	0.9582	970.6897	721	0.9952
24–28 cm	5.8846	0.9635	889.9385	664	0.9919
28–32 cm	5.7551	0.9601	1032.8364	730	0.9985
32–36 cm	5.6039	0.9563	983.8399	698	0.9973
36–40 cm	5.6640	0.9604	905.8141	630	0.9924
40–44 cm	5.3314	0.9352	810.8497	577	0.9945
44–48 cm	5.3838	0.9205	882.1759	650	0.9934
48–52 cm	5.0336	0.9071	807.2265	596	0.9911
52–56 cm	4.6489	0.8604	742.9760	525	0.9959
56–60 cm	5.0886	0.9150	764.6305	548	0.9909
60–64 cm	4.4689	0.8776	764.2769	516	0.9903
64–68 cm	4.9358	0.9036	778.4738	517	0.9928
68–72 cm	3.9983	0.7908	675.0231	465	0.9909
72–76 cm	4.7031	0.8939	754.0279	514	0.9947
76–80 cm	4.5591	0.8826	648.8547	449	0.9918
80–84 cm	4.2680	0.8085	572.1768	414	0.9968
84–88 cm	4.6890	0.8619	639.4648	467	0.9925
88–92 cm	4.5486	0.8530	703.2688	484	0.9958
92–96 cm	5.0342	0.9157	782.2206	542	0.9978
96–100 cm	5.0927	0.9045	898.1468	611	0.9899

## Data Availability

All raw sequence data obtained in this study have been deposited at the Sequence Read Archive database of the National Center for Biotechnology Information under accession number PRJNA853917. These data are publicly accessible at http://www.ncbi.nlm.nih.gov/.
